# Characterization of digital annular pulleys and their entheses: an ultrasonographic study with anatomical and histological correlations

**DOI:** 10.1093/rheumatology/kead614

**Published:** 2023-11-23

**Authors:** Luis Coronel, Peter Mandl, Maribel Miguel-Pérez, Joan Blasi, Maria Antonietta D’Agostino, Carlo Martinoli, David A Bong, Ingrid Möller

**Affiliations:** Department of Rheumatology, Vall d’Hebron Hospital Universitari, Vall d’Hebron Barcelona Campus, Barcelona, Spain; Unit of Human Anatomy and Embryology, Department of Pathology and Experimental Therapeutics, Faculty of Medicine and Health Sciences (Bellvitge Campus), University of Barcelona, Barcelona, Spain; Instituto Poal de Reumatología, Barcelona, Spain; Department of Rheumatology, Medical University of Vienna, Vienna, Austria; Unit of Human Anatomy and Embryology, Department of Pathology and Experimental Therapeutics, Faculty of Medicine and Health Sciences (Bellvitge Campus), University of Barcelona, Barcelona, Spain; Histology Unit, Faculty of Medicine and Health Sciences (Bellvitge Campus), University of Barcelona, Barcelona, Spain; Division of Rheumatology, Università Cattolica del Sacro Cuore, Policlinico Universitario Agostino Gemelli IRCSS, Rome, Italy; Department of Health Sciences, University of Genoa, Genoa, Italy; Instituto Poal de Reumatología, Barcelona, Spain; Unit of Human Anatomy and Embryology, Department of Pathology and Experimental Therapeutics, Faculty of Medicine and Health Sciences (Bellvitge Campus), University of Barcelona, Barcelona, Spain; Instituto Poal de Reumatología, Barcelona, Spain

**Keywords:** spondyloarthropathies (including psoriatic arthritis), hand, histopathology, ultrasonography, diagnostic imaging

## Abstract

**Objectives:**

Digital annular pulleys (DAPs) are important anatomical structures for finger function. The anatomy, histology and imaging assessment of DAPs, particularly at the level of their entheses, are still not clearly defined. The advent of high-frequency US transducers opened new perspectives in evaluating submillimetre-scale structures, such as pulleys, paving the way for their global assessment. The study aimed to characterize DAPs from an anatomical, histological and US perspective, focusing on the detection and complete description of pulley entheses.

**Methods:**

US assessment and gross anatomy dissection were conducted on 20 cadaveric hands to study DAP thickness and structure, including enthesis identification. The results of the US and anatomical measurements were correlated. DAP entheses identified by US were characterized via histological analysis. DAPs in 20 healthy controls (HCs) were detected and measured by US. The A1, A2 and A4 DAP entheses were assessed using a new dynamic manoeuvre to better evaluate those structures.

**Results:**

A total of 1200 DAPs (400 cadaveric, 800 HCs) were analysed. The cadaveric study demonstrated strong correlation between anatomical and US measurement of DAPs (*r* = 0.96). At the histological level, DAP entheses at the volar plate, sesamoid bones or phalangeal ridges contained fibrous and fibrocartilaginous tissue. US assessment of A1, A2 and A4 DAPs in HCs allowed the identification of 718/720 (99.73%) entheses.

**Conclusion:**

US is an effective tool to detect and study DAPs. DAP entheses reveal both fibrous and fibrocartilaginous characteristics. A newly described manoeuvre to optimize DAP enthesis visualization enhances their detection by US.

Rheumatology key messages

- Histological examination of DAP insertions revealed their entheseal composition, including both fibrous and fibrocartilaginous tissue.
- High-frequency US is a reliable tool to study DAPs and their entheses.
- A newly described dynamic manoeuvre allows better exposure of DAP entheses.

## Introduction

The digital annular pulleys (DAPs) of the hand are part of a complex structure required to guarantee optimal function of the flexor tendons by preventing their bowstringing, as well as ulnar and radial displacement, through the formation of efficient tendon gliding tunnels [1]. Except for the thumb, there are five DAPs, numbered from A1 to A5, running from the proximal to the distal part of each finger. A1, A3 andA5 lay over the MCP, PIP and DIP joints, respectively. They have a thin, flexible structure and insert mainly into the volar plate. A2 and A4 are located above the proximal and middle phalanges, being broader and thicker with direct insertions into bone [2, 3].

As described by Doyle [1], pulleys are fibrous tissue condensations that constitute the reticular part overlaying the synovial component of the flexor tendon sheath. Histologically, pulleys display a three-layered structure composed of an outer layer, continuous with the membranous tendon sheath, which is vascularized, thus providing nutrition to the reticular structure; the middle layer, where transversely oriented collagen fibrils and interspersed spindle-shaped fibroblasts are located to grant resistance to biomechanical forces generated during finger flexion; and the inner layer, composed of longitudinally oriented collagen fibrils and hyaluronic acid–producing fibroblasts, necessary to avoid friction and allow gliding of the underlying tendon. At this level, the presence of chondrocyte-like cells and scattered synovial cells has been depicted with contrasting results [4, 5].

Intriguingly, no clear description of pulley attachment sites is available thus far. The A2 and A4 DAPs directly insert into the phalangeal shaft [6] and at this level bony ridges appear with aging. These insertions have been suggested to behave as anatomical entheses with a transition zone consisting of fibrocartilage [7]. Less is known regarding A1, A3 and A5, where the anatomy is more complex due to the presence of joint capsules, volar plates and, variably, sesamoid bones. Recently, pulleys have attracted interest in inflammatory processes of arthritis, emphasizing their role as functional entheses going beyond their unique mechanical function [8].

*In vivo* studies of pulleys can be feasibly performed with high-frequency US transducers [9, 10], as hockey stick US probes of at least 18 MHz frequency, owing to their superior resolution, enable real-time assessment of superficial structures and thus add important anatomical and physiological information to what is observed in cadaver-based studies and without the need for dissection and surgical exposure of tissues.

Considering the current state of knowledge of DAP anatomy and its visualization by US, we conducted the present study with a 3-fold objective: to comprehensively depict DAP morphology and histology using human specimens; to introduce a landmark-based US scanning technique allowing the systematic examination of these structures, including the evaluation of their entheses and attachments; and to verify its application in healthy control (HC) subjects.

## Methods

The study was conducted in accordance with the Declaration of Helsinki and approved by the Bioethics Committee of the University of Barcelona (IRB00003099). Informed consent was obtained from HCs who underwent a US examination of DAPs.

## Finger pulley identification by US

A LOGIQ P9 US unit (GE Ultrasound Korea, Seongnam, Republic of Korea) equipped with an 8–18 MHz hockey stick transducer and an Aplio i800 Prism Edition (Canon Medical Systems, Tustin, CA, USA) US unit equipped with an 8–22 MHz hockey stick transducer were used for DAP assessment on cadaveric specimens and HCs, respectively.

All examinations were performed by the same investigator (L.C.) with 6 years of experience in musculoskeletal US and a reliability exercise was performed with a senior musculoskeletal sonographer (I.M.) with >30 years of experience and one of the responsible teachers of the sonoanatomy program at the University of Barcelona.

### US study of DAPs in cadavers

The US study was conducted on 80 fingers of 20 hands (12 right, 8 left) from adult corpses (11 women, 9 men) cryopreserved at −20°C in the Dissection Laboratory of the Faculty of Medicine and Health Sciences at the University of Barcelona. All the specimens were donations to the Faculty of Medicine and Health Science. They did not present evidence of traumatic injuries or surgical scars. The samples were coded with a univocal number to allow identification according to the dissection order and the sonographic examination.

The thickness of each pulley, from A1 to A5 of the second, third, fourth and fifth fingers, was measured twice at their thickest site both in transverse and longitudinal views (Supplementary Fig. S1, available at *Rheumatology* online) and the mean value was considered as a final result, as previously described [11]. Eight fingers were evaluated consecutively by two sonographers (L.C. and I.M.), who were blinded with respect to the examination performed by the other sonographer, in order to assess intra- and interobserver reliability.

The entheses of A1, A2 and A4 were assessed in B mode in a transverse scan at both the ulnar and the radial level and scored as absent/present (0/1); A3 and A5 were excluded from this analysis because of the poor visualization of their insertions. The landmarks used to identify pulley entheses were the distal palmar fold, volar plate and sesamoids for A1; the proximal phalanx ridges and the exit of the Camper’s chiasm for A2; and the middle phalangeal ridges and the insertion of the flexor digitorum superficialis tendon for A4 [7] (Supplementary Table S1, available at *Rheumatology* online).

A new dynamic manoeuvre was adopted to overcome technical difficulties due to anisotropy in detecting pulley entheses of A2 and A4. After obtaining the optimal visualization of the pulley in a transverse scan, a composite movement, consisting of the simultaneous rotation of the finger coupled with sliding of the probe 30–45 degrees in the opposite direction, was performed to ensure the perpendicular insonation of the enthesis (contralateral rotation manoeuvre, Supplementary Videos S1 and S2, available at *Rheumatology* online). To identify the A1 enthesis, the probe was positioned slightly oblique with respect to the longitudinal axis of the finger.

### US study of DAPs in HCs

A total of 20 HCs were recruited at the Rheumatology Unit of the Vall d’Hebron Hospital, Barcelona. DAPs from A1 to A5 in the second through fifth fingers were examined.

During scanning, individuals were seated in front of the examiner with the hand resting on the examination table in a supine position with fully extended fingers. Pulleys were assessed bilaterally in both longitudinal and transverse scans, as indicated by the 2017 EULAR standardized procedures for US imaging in rheumatology [12]. During passive flexion–extension of the finger, a dynamic assessment was performed to distinguish the static pulleys from the mobile tendons that move with the fingers. The technique described in the previous section was applied to assess pulley thickness and entheses.

All dynamic procedures were recorded as videos and sequential static images of each examined structure were obtained.

### Anatomical study of DAPs

In the cadaveric specimens, a needle was inserted in each pulley detected by US before dissection. Dissection was performed from the mid-carpal area to the distal end of each finger, second to fifth; thumbs were excluded [13, 14]. The skin and soft tissues around the tendon sheath were removed and the pulleys were exposed and identified using a surgical microscope. Each pulley was measured twice at the point of maximal thickness with a Vernier caliper (absolute Solar Caliper Series 500, Mitutoyo, Aurora, IL, USA), considering the mean value as the final result. Twenty DAPs of four fingers were measured consecutively by L.C. and M.M., blinded to each other, in order to obtain intra- and interobserver reliability.

### Histological study of DAPs

Paraffin-embedded sections of eight fingers at the pulley level, less than 8 × 5 mm in length and width, were obtained. The tissue was decalcified using EDTA, sectioned by microtome in the transverse axis, fixed in buffered formalin for 2 days and stained with haematoxylin and eosin [15]. Entheses appeared as fibrous or fibrocartilaginous tissue in the transition zone between DAPs and bones/volar plates.

The entheses of A1, A2 and A4 were assessed at both the ulnar and radial sides and the presence of fibrocartilage at the entheses was scored as absent/present (0/1). Each enthesis was evaluated and the histological description of the tissue structure as fibrous, fibrocartilaginous or mixed at the insertional level was reported (Supplementary Table S2, available at *Rheumatology* online).

All preparations were observed with a DMD 108 microscope (Leica Microsystems, Deerfield, IL, USA) at 10× magnification.

### Statistical analysis

Quantitative demographic variables were summarized as means and s.d.s and qualitative variables as frequencies and percentages. Variables were compared across groups using independent samples, Student’s *t*-test and χ^2^.

The intraclass correlation coefficient (ICC) with 95% CIs was calculated to evaluate intrarater and interrater reliability for repeated DAP measurements with US and digital caliper. Reliability was qualified with the ICC as bad or null (ICC < 0.20), poor (ICC = 0.21–0.40), moderate (ICC = 0.41–0.60), good (ICC = 0.61–0.80) or very good (ICC = 0.81–1.00).

All tests were two-sided and *P*-values <0.05 were considered statistically significant. Statistical analyses were performed using RStudio version 2022.12.0 + 353 (Posit Software, Boston, MA, USA).

## Results

### Demographic and clinical features

The mean age of donors and HCs was 81 years (s.d. 9; range 58–96) and 47 years (s.d. 15; range 21–74), respectively (*P* < 0.001). Additional demographic data such as manual work, playing hand sports, BMI and hand dominance was available only for HCs. Specifically, two were manual workers and seven practiced hand sports. The mean BMI was <25 and all participants were right-handed. Table 1 shows the demographic and clinical characteristics of the two cohorts.

**Table 1.** Demographic characteristics of cadavers and HCs

Characteristics	Cadavers (*n *=* *20)	HCs (*n *=* *20)	*P*-value
Age, years, mean (s.d.)	81 (9)	47 (15)	<0.001
Female, *n* (%)	11 (55)	15 (75)	0.236
BMI, kg/m^2^, mean (s.d.)	–	23.1 (3.2)	
Manual workers, *n* (%)	–	2 (10)	
Hand sports, *n* (%)	–	7 (35)	
Right-handed, *n* (%)	–	20 (100)	

### Assessment of pulley thickness in cadavers and HCs

A total of 1200 pulleys (800 in HCs, 400 in cadavers) were assessed by US and 400 by anatomical dissection. The mean values of DAP thickness detected by US were analysed for each finger from the second to fifth in both specimens and HCs.

In DAPs of HCs, the mean thickness by US was lower than in cadavers, with statistically significant differences for A1 thickness in the second, third and fourth fingers; A2 in all fingers; and A4 in the third, fourth and fifth fingers (*P* < 0.05). Detailed results are presented in Table 2.

**Table 2.** Pulley thickness for all DAPs (A1–A5) in fingers 2–5 in cadavers and HCs measured by US

DAPs, mm, mean (s.d.)	Cadavers				HC			
	2	3	4	5	2	3	4	5
A1	0.353* (0.066)	0.395* (0.083)	0.378* (0.073)	0.295 (0.09)	0.300 (0.055)	0.348 (0.073)	0.316 (0.065)	0.284 (0.093)
A2	0.413* (0.072)	0.455* (0.084)	0.408* (0.086)	0.328* (0.075)	0.358 (0.092)	0.347 (0.082)	0.350 (0.087)	0.255 (0.052)
A3	0.135 (0.043)	0.135 (0.056)	0.145 (0.051)	0.128 (0.044)	0.141 (0.054)	0.140 (0.058)	0.131 (0.046)	0.131 (0.046)
A4	0.373 (0.072)	0.383* (0.075)	0.358* (0.075)	0.313* (0.086)	0.333 (0.082)	0.331 (0.060)	0.293 (0.085)	0.255 (0.058)
A5	0.106 (0.024)	0.113 (0.034)	0.113 (0.033)	0.103 (0.013)	0.104 (0.023)	0.105 (0.023)	0.104 (0.025)	0.102 (0.030)

* *P* < 0.05, indicating statistically significant thicker DAPs in cadavers *vs* HCs.

**Figure 1.**
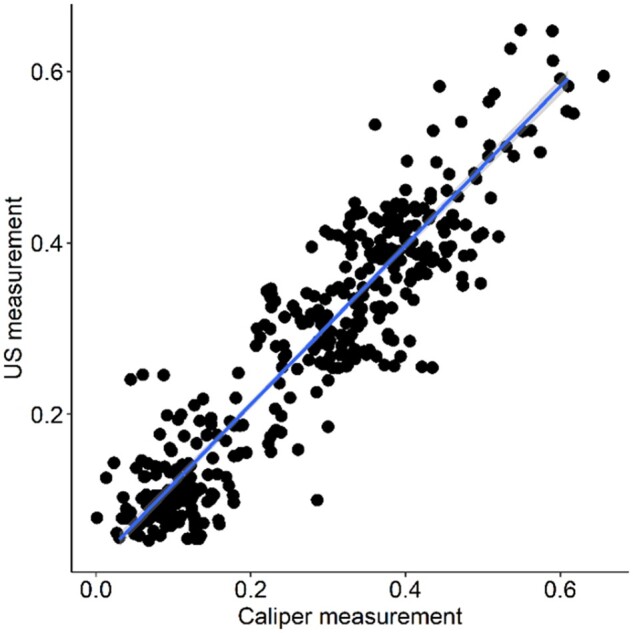
Correlation (*r*=0.96) between US and digital caliper measurements of DAPs

The results of the DAP US assessment in cadavers were confirmed in the anatomical study (Table 3). No significant differences between the US and the caliper measurements were noted. Thus a positive strong correlation (*r* = 0.96) between US and caliper measurements of DAPs in specimens was found (Fig. 1).

**Table 3.** Digital caliper measurements of DAP thickness in cadavers

DAPs, mm, mean (s.d.)	II	III	IV	V
A1	0.348 (0.065)	0.395 (0.081)	0.358 (0.075)	0.290 (0.086)
A2	0.389 (0.082)	0.459 (0.086)	0.400 (0.078)	0.325 (0.089)
A3	0.125 (0.047)	0.132 (0.057)	0.128 (0.028)	0.121 (0.041)
A4	0.378 (0.081)	0.393 (0.073)	0.371 (0.088)	0.315 (0.087)
A5, mean (SD), mm	0.083 (0.021)	0.095 (0.023)	0.086 (0.018)	0.083 (0.026)

A total of 2.75% (11/400) of pulley measurements in the specimen group were lost because of technical difficulties related to cryopreservation and possible alteration of tissue. In particular, four pulleys were not detected by US but were identified in the dissection study, while seven pulleys were evident only on US.

No significant correlation was found between demographic data and pulley thickness.

The ICC for the intrarater reliability for DAP measurements by US was 0.83 (95% CI 0.707, 0.921) and by digital caliper was 0.91 (95% CI 0.789, 0.963). The ICC for the interrater reliability by US was 0.859 (95% CI 0.756, 0.933) and by digital caliper was 0.874 (95% CI 0.760, 0.943).

### DAP entheses evaluation by anatomy, histology and US

**Figure 2.**
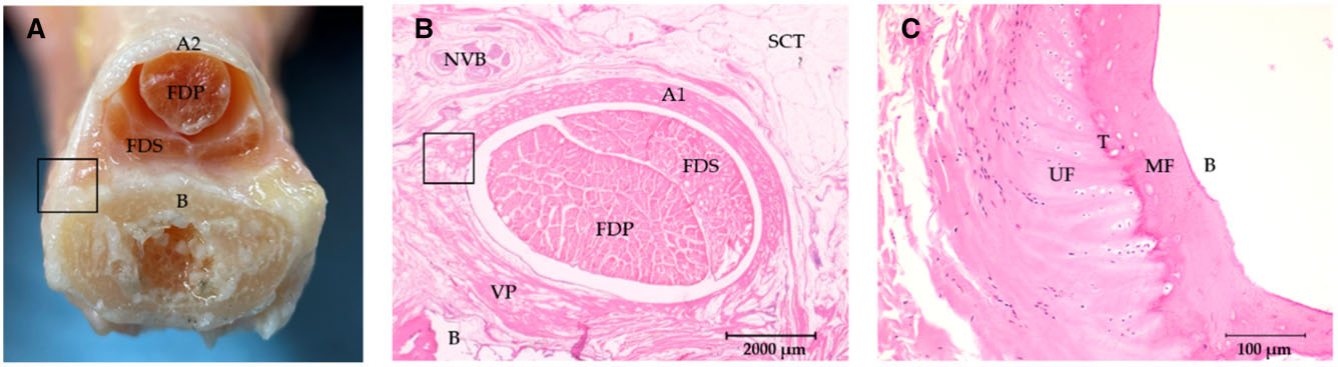
Anatomy and histology of DAPs. **(A)** Anatomical dissection of A2 pulley and third finger showing the insertion of the pulley into the bony ridge of the proximal phalanx (box). **(B)** Histology of the third finger A1 pulley (H&E) showing the insertion of the pulley into the volar plate (box). **(C)** Histology of the third finger A2 fibrocartilaginous enthesis at the bony ridge (H&E). B: bone; FDP: flexor digitorum profundus; FDS: flexor digitorum superficialis; H&E: haematoxylin and eosin staining; MF: mineralized fibrocartilage; NVB: neurovascular bundle; SCT: subcutaneous tissue; UF: unmineralized fibrocartilage; T: tidemark; VP: volar plate

The anatomical (Fig. 2A) and histological studies demonstrated the presence and composition of DAP entheses. Entheses are composed of mixed fibrous tissue with fibrillary extensions that can pass through the tidemark to join the bone or fibrocartilaginous tissues beneath. Description of the main characteristics of the different DAP entheses are noted in Supplementary Table S2, available at *Rheumatology* online.

We found that the A1 entheses are connected to the volar plate, a fibrocartilaginous structure renowned for its attachment to the bone at the metacarpal level consisting of an enthesis into fibrocartilage. In the presence of sesamoid bones, we found that the DAP entheses exhibit expansions by which they attach to the sesamoids before anchoring to the volar plate (Fig. 2B).

The A2 and A4 entheses, because of their anatomical position, were found to join bone at the level of bony ridges on the proximal and middle phalangeal shafts (Fig. 2C).

**Figure 3.**
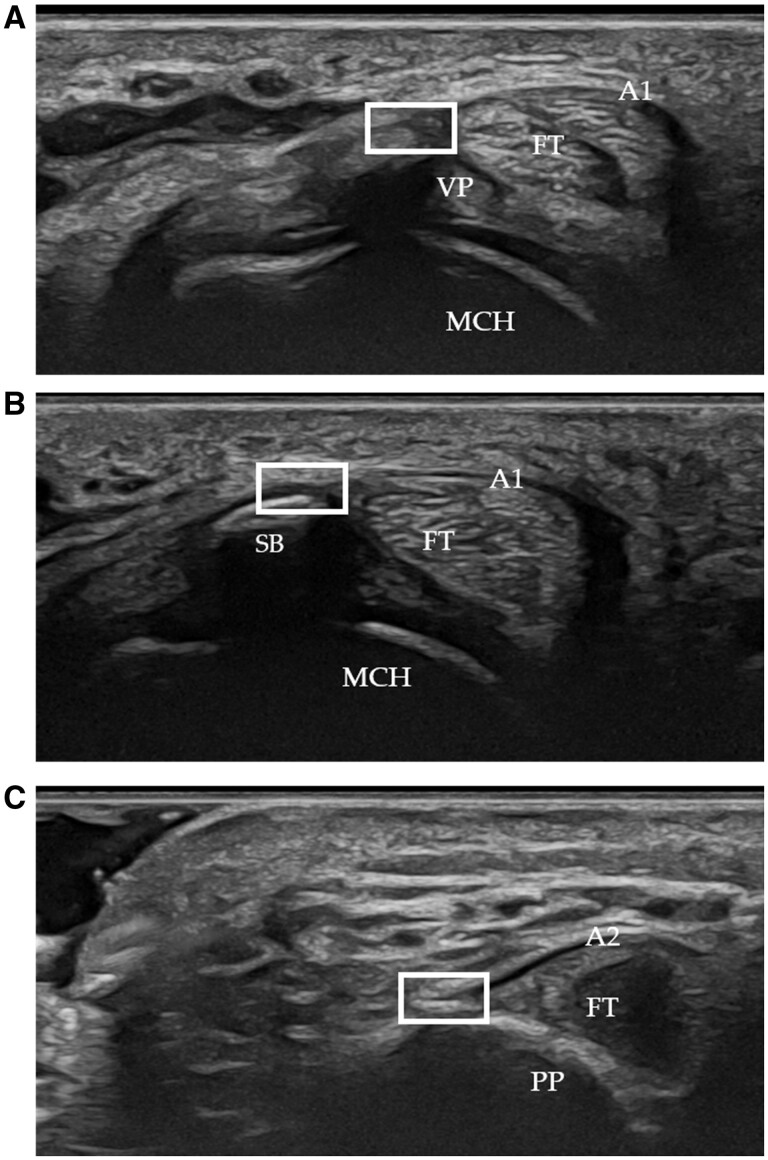
Transverse US scan of the second finger showing a longitudinal image of A1 and A2 DAP entheses (white box). **(A)** A1 pulley enthesis at the volar plate. **(B)** A1 pulley entheseal expansion to the sesamoid bone. **(C)** A2 enthesis at the bony ridge of the proximal phalanx. FT: flexor tendon; MCH: metacarpal head; PP: proximal phalanx; SB: sesamoid bone; VP: volar plate

By US evaluation, 718/720 (99.73%) of the pulley entheses, considering both specimens and HCs, were detected with the manoeuvre proposed in the Methods section. Only 0.27% (2/270) were undetectable. A1 entheses were evidenced at their insertion into the volar plate (Fig. 3A), with additional attachments to sesamoid bones at the second and fifth fingers (Fig. 3B), corroborating the anatomical data. Notably, 23.8% (81/340) of A1 entheses involved sesamoid bones in the second [43.2% (35/81)] and fifth fingers [56.8% (46/81)], which were exclusively located in the radial aspect of the second finger and almost exclusively in the ulnar aspect of the fifth finger.

The A2 (Fig. 3C) and A4 entheses were visualized at the ulnar and radial aspects of the proximal and middle phalangeal bones, respectively.

## Discussion

The DAP system has fundamental biomechanical properties that facilitate fingers to perform delicate and precise motor tasks [3]. Additionally, DAPs are emerging as critical anatomical structures involved in various rheumatic diseases, especially PsA [10]. In this regard, the main aim of this study was to fully elucidate their anatomy, with a particular focus on their entheses, and to describe a new scanning technique for their optimal assessment by US.

The first parameter assessed was DAP thickness, which was significantly greater in cadaveric fingers as compared with HCs. The difference may be related to age variations between the two cohorts. Previous studies have demonstrated that DAP thickness increases with age, perhaps due to the continuous chronic stimulation on these structures [10, 16]. In this regard, a cut-off value of 1 mm in thickness has been proposed for A1 as the possible threshold to support the diagnosis of trigger finger [17]. In contrast, no clear cut-off values have been determined for inflammatory conditions [11].

In line with the literature data, A2 appeared as the thickest DAP, followed by A1 and A4 [13]. We demonstrated that US is a valuable tool to study DAPs, capable of demonstrating them in almost all cases. Furthermore, we confirmed US as a consistent tool for measuring DAP thickness.

Owing to their size, A1, A2 and A4 are easy to evaluate by US, and despite high variability in the pattern appearance of the DAP system, they are consistently present [13]. Bearing this in mind, we focused our attention on the entheses of these pulleys and assessed them via anatomical, histological and US imaging for the first time. Two types of entheses have been described: anatomical and functional entheses. Anatomical entheses are those where tendons, ligaments, fascia and joint capsules attach to bone, whereas functional entheses occur where tendons or ligaments wrap around bony pulleys. Microscopically, the transition from soft to hard tissue is depicted either as a fibrous or a fibrocartilaginous interface [18]. DAP entheses fit both definitions and are essential for transmitting and dissipating contractile forces to tendons and bones at the finger level. Our analysis of A1, A2 and A4 demonstrating the presence of fibrous and fibrocartilaginous tissue is in agreement with previous literature [7].

Enthesitis plays a pivotal role in the development of inflammatory diseases such as SpA and PsA. In particular, DAP functional enthesitis has been indicated in dactylitis [14] and we suggest that involvement of the anatomical enthesis may also play a role. To date, most of the studies on DAPs were performed using MRI [19, 20]. In our study, we demonstrate that US can visualize the A1, A2 and A4 anatomical entheses. Notably, use of a newly described manoeuvre showed visualization of DAP entheses in >95% of cases, in contrast with the lower percentage reported previously, especially for A4 [19].

The A1 anatomical enthesis inserts into the volar plate and sesamoid bones in the second and fifth fingers [21]. A2 and A4 entheses attach to the phalanx shaft, with A4 presenting a more complex structure because of its close relation to the flexor superficialis tendon in its enthesis. The bony prominences into which A2 and A4 insert are known as ridges. Ridges increase with aging and present enthesophytes-like characteristics as they derive from new bone apposition in sites exposed to high mechanical stress [7, 19]. Such behaviour reinforces the importance of DAP entheses in inflammatory conditions. In the specific case of pulleys, increased thickness and vascularization are elements that characterize changes in the functional enthesis in SpA [14].

The implications of our research in clinical practice are multiple: new landmarks, such as bony ridges and sesamoids, to identify finger entheses may help radiologists, rheumatologists and surgeons deal with such structures. In addition, US is an inexpensive, readily available and repeatable imaging technique unassociated with ionizing radiation. DAP enthesis evaluation has the potential to be an important component of the initial assessment and follow-up of patients with inflammatory arthritis.

Some limitations of the present study should be mentioned. First, the age gap between donors and HCs could be an essential factor affecting DAP thickness and structure. Aging could contribute to DAP thickness changes due to chronic mechanical exposure [10] or at its entheses [7], so our specimens may not precisely mirror the anatomy of DAP in younger people. During the preparation of the tissue samples for histological analyses, some data were not available due to the processing methods, staining, cutting and thawing, but the samples were adequate to answer the initial hypothesis. Obtaining tissue samples of DAPs from healthy individuals is very tricky, and even the hypothesis of performing biopsies of such structures, as already done for large-size entheses, is not a viable option [22]. In this regard, the evidence that US can accurately assess DAP thickness and entheses is a significant strength of our research. In addition, the use of US proposed herein needs to be further evaluated for reproducibility and reliability on larger cohorts of subjects.

Once we have defined the gross features of DAPs and their entheses in healthy conditions, we aim to address new questions regarding their alterations in mechanical and inflammatory diseases. The involvement of anatomical DAP entheses in dactylitis and SpA (dactylitis as a more severe phenotype) will be an interesting future research topic.

In conclusion, our study highlighted the entheseal architecture of A1, A2 and A4, which contain a mixture of fibrous and fibrocartilaginous tissue. Moreover, high-frequency US was proven to be an effective tool for evaluating DAP thickness and dynamically assessing DAP entheses, particularly when the newly proposed contralateral rotation manoeuvre is performed.

## Supplementary Material

kead614_Supplementary_Data

## Data Availability

The data underlying this article are available in the article and in its online supplementary material.
