# Supplementary material for: Characterization of digital annular pulleys and their entheses: an ultrasonographic study with anatomical and histological correlations
Source: Rheumatology (Oxford). 2023 Nov 23;63(11):3050–5. doi: 10.1093/rheumatology/kead614 (PMC11534144; doi:10.1093/rheumatology/kead614)
Supplement: kead614_Supplementary_Data [file kead614_supplementary_data.zip › kead614_Supplementary_Data/rhe-23-1581-File006.docx]

**Supplementary Table S1.** Probe positioning for identifying DAP entheses

| DAP | Anatomical reference | Probe position | US reference | US image |
| --- | --- | --- | --- | --- |
| A1 | Distal palmar fold, MCP joint | 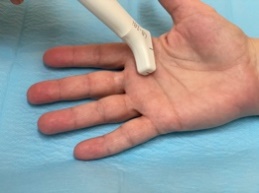 | Volar plate and sesamoid bones* | 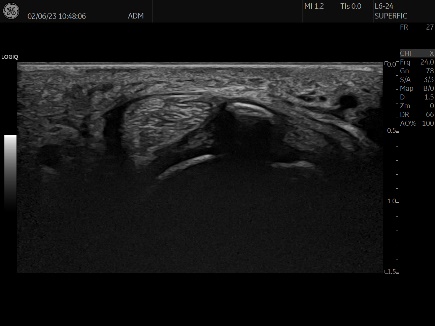  **^**  FT  VP  S |
| A2 | Midpoint of the proximal phalanx | 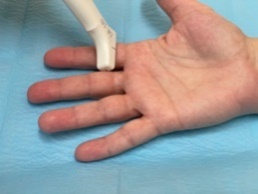 | Phalangeal ridges and the exit of Camper’s chiasm | 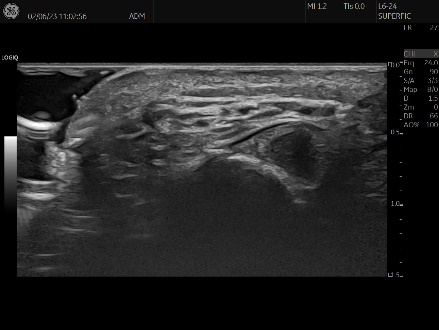  **^**  FT  R  PP |
| A4 | Midpoint of the middle phalanx | 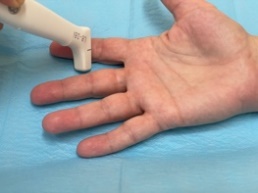 | Phalangeal ridges and the insertion of the flexor digitorum superficialis muscle | 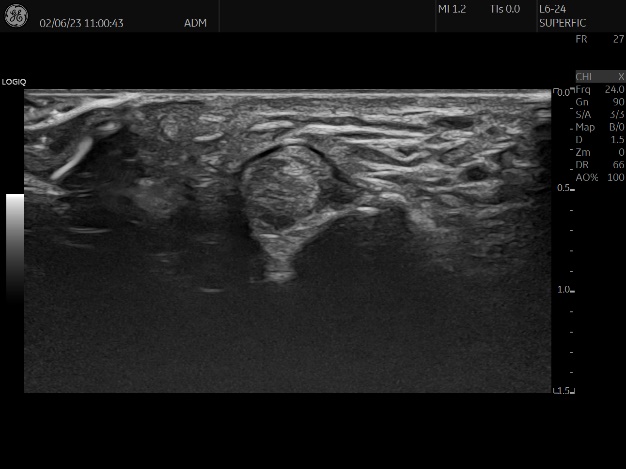  **^**  FT  R  MP |

* More frequently identified in the radial side of the 2^nd^ finger and the ulnar side of the 5^th^ finger at the metacarpophalangeal level.

DAP: Digital annular pulley, FT: Flexor tendons, MCP: Metacarpophalangeal, MP: Middle phalanx, PP: Proximal Phalanx, R: Ridge, S: Sesamoid bone, VP: Volar plate, ^: Enthesis.
