# Supplementary material for: Characterization of digital annular pulleys and their entheses: an ultrasonographic study with anatomical and histological correlations
Source: Rheumatology (Oxford). 2023 Nov 23;63(11):3050–5. doi: 10.1093/rheumatology/kead614 (PMC11534144; doi:10.1093/rheumatology/kead614)
Supplement: kead614_Supplementary_Data [file kead614_supplementary_data.zip › kead614_Supplementary_Data/rhe-23-1581-File007.docx]

**Supplementary Table S2.** Histological characteristics of DAP

|  | **Presence of fibrocartilage at the enthesis** | | | **Description of the enthesis** |
| --- | --- | --- | --- | --- |
|  | Yes | No | Not available |  |
| **A1** | 11 | 3 | 2 | Fibrous and Fibrocartilaginous enthesis (volar plate/sesamoid bone) |
| **A2** | 11 | 1 | 4 | Fibrous and Fibrocartilaginous enthesis |
| **A4** | 9 | 2 | 5 | Fibrous and Fibrocartilaginous enthesis |

2 different corpses, 2 hands (1 right/1 left), 1 female, 1 male, 8 fingers, 24 pulleys

Not available: data lost due to cutting, sample preparation, staining and processing
