# Supplementary figures and images for: Characterization of digital annular pulleys and their entheses: an ultrasonographic study with anatomical and histological correlations
Source: Rheumatology (Oxford). 2023 Nov 23;63(11):3050–5. doi: 10.1093/rheumatology/kead614 (PMC11534144; doi:10.1093/rheumatology/kead614)

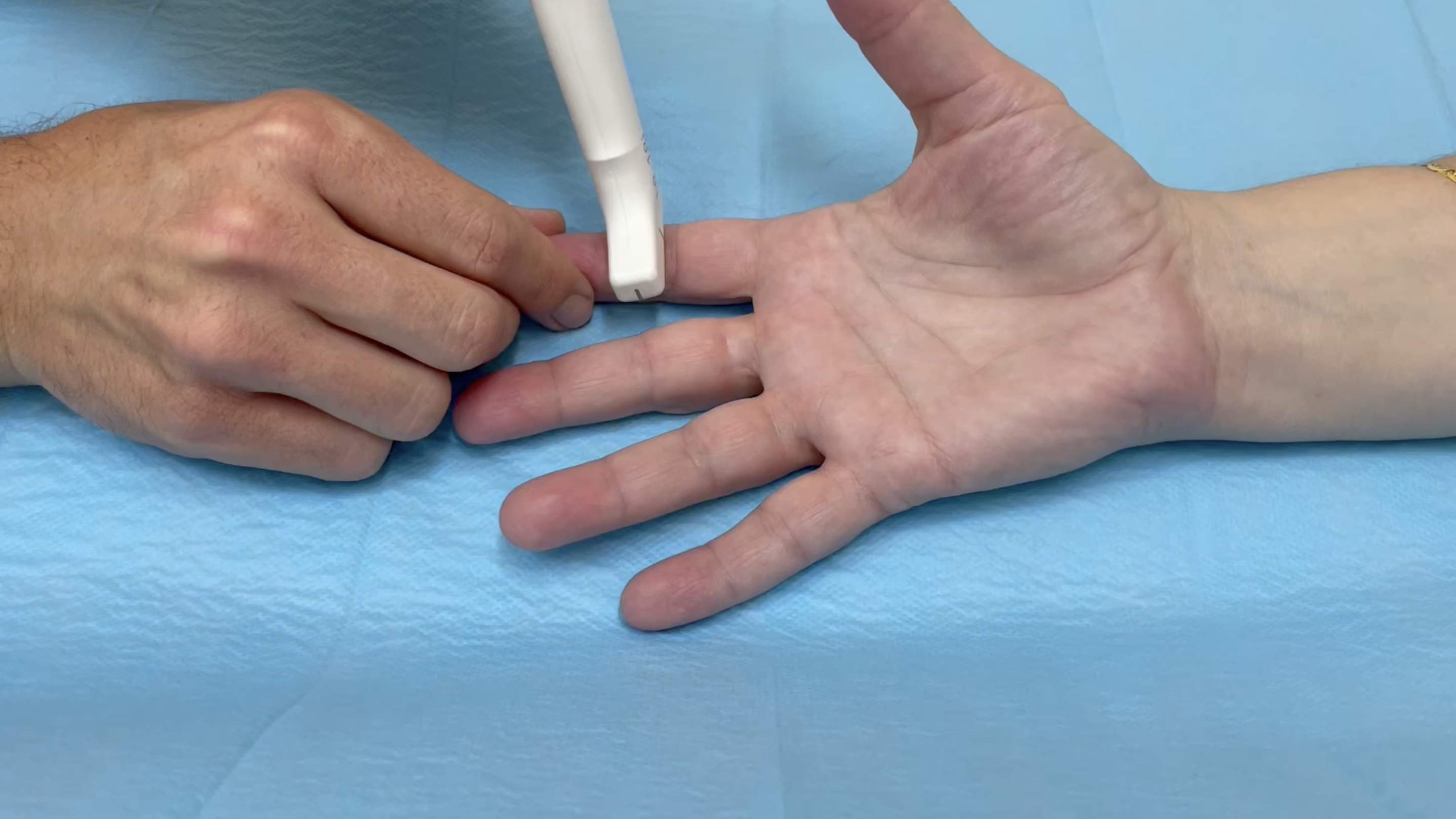

Supplement: kead614_Supplementary_Data [file kead614_supplementary_data.zip › kead614_Supplementary_Data/rhe-23-1581-File009.jpg]

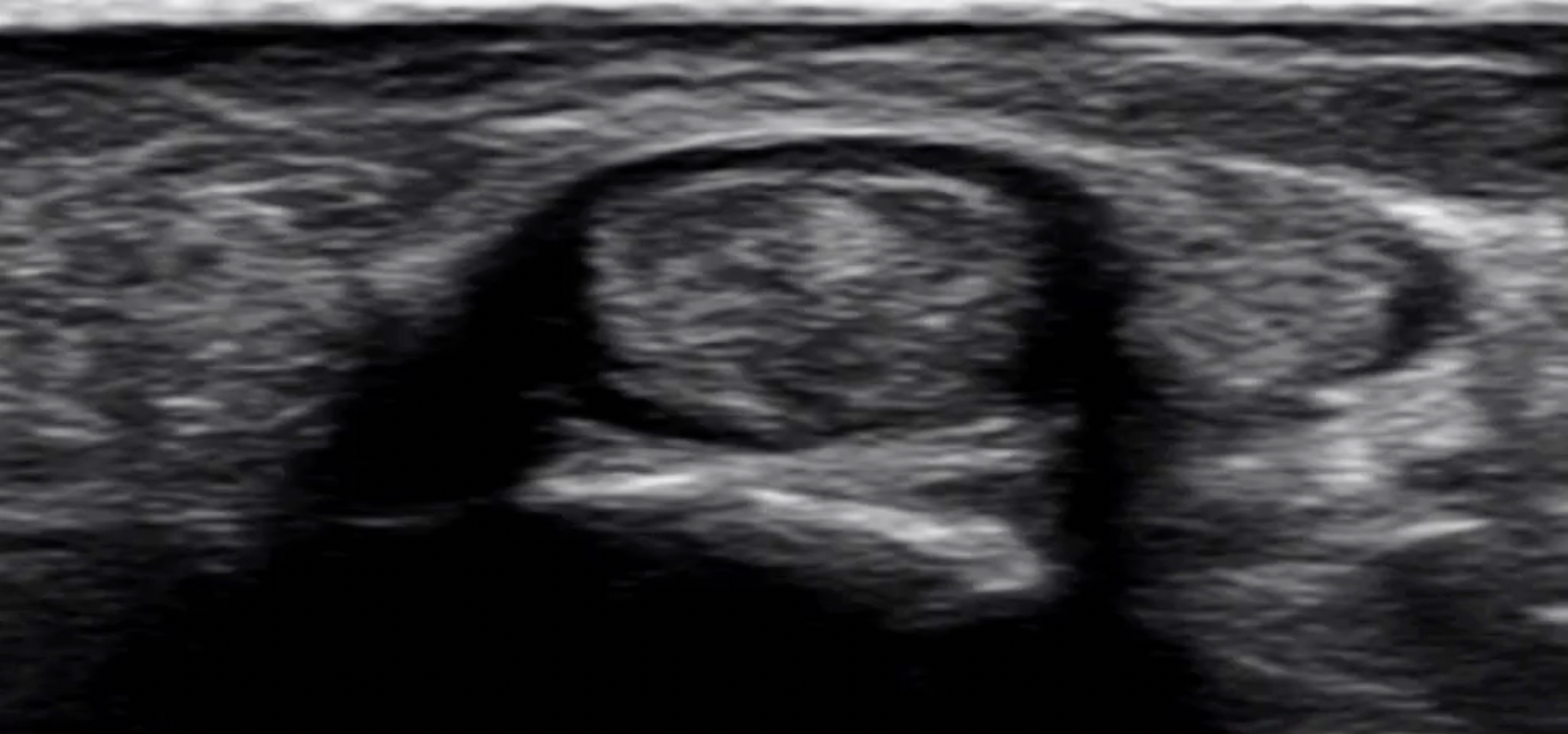

Supplement: kead614_Supplementary_Data [file kead614_supplementary_data.zip › kead614_Supplementary_Data/rhe-23-1581-File011.jpg]
